# Cumulative thalidomide exposure, peripheral neuropathy, and clinical outcomes in pediatric and very early-onset inflammatory bowel disease: a retrospective cohort study

**DOI:** 10.1016/j.clinsp.2026.101093

**Published:** 2026-09-08

**Authors:** Jing Mao, Pan Li, Zebin Chen, Zhongqiang Cao, Ling Yang, Zhuyin Li, Zhaoxia Wang, Shuo Liang

**Affiliations:** a Department of Pharmacy, Shenzhen Children's Hospital, Shen Zhen, China; b Cancer Institute, School of Medicine, Jianghan University, Wuhan, China; c Department of Gastroenterology, Shenzhen Children's Hospital, Shenzhen, Guangdong, China; d Shenzhen Polytechnic University, Shenzhen, China; e Hubei Provincial Demonstration Center for Experimental Medicine Education, School of Medicine, Jianghan University, China

**Keywords:** Thalidomide, Pediatric inflammatory bowel disease, Cumulative dose, Peripheral neuropathy, Very early-onset IBD

## Abstract

**Background:**

Thalidomide serves as a rescue therapy for refractory pediatric Inflammatory Bowel Disease (pIBD), but its use is constrained by the risk of thalidomide-induced Peripheral Neuropathy (TiPN). The quantitative relationship between cumulative dose and TiPN, as well as the risk profile in the specific population of Very Early-Onset IBD (VEOIBD), lacks precise evaluation.

**Methods:**

We conducted a single-center, retrospective cohort study of children with IBD treated with thalidomide from 2018 to 2025. We compared cumulative thalidomide doses between TiPN and non-TiPN groups and employed ROC curve analysis to determine a predictive dose threshold. The cohort was further stratified into VEOIBD and pIBD groups to compare dynamic inflammatory marker changes and overall drug tolerability.

**Results:**

Among 49 included patients, the incidence of TiPN was 26.5% (13/49). The cumulative thalidomide dose was significantly higher in the TiPN group (median 19.5 g vs. 8.35 g, p = 0.008). A cumulative dose threshold of > 15.38 g predicted TiPN with an AUC of 0.756 (sensitivity 0.722, specificity 0.692). While VEOIBD patients (n = 14) had a distinct disease treatment history, they experienced significantly fewer overall adverse events than pIBD patients (3/14 vs. 19/35, p = 0.04), with no significant difference in TiPN incidence.

**Conclusions:**

Cumulative thalidomide dose is a strong predictor of TiPN in children with IBD. Despite being a more refractory population, VEOIBD patients demonstrated better overall tolerance to thalidomide. Monitoring cumulative dose is crucial for mitigating neurotoxicity and optimizing thalidomide therapy in pIBD.

## Highlights

• First study evaluating thalidomide neurotoxicity in pediatric VEOIBD patients.
• Cumulative dose modeled as a continuous variable for precise risk prediction.
• A critical cumulative dose threshold of 15.38 g was identified for TiPN risk.
• VEOIBD showed paradoxically better tolerability despite refractory disease.

## Introduction

Inflammatory Bowel Disease (IBD) is a chronic, relapsing intestinal disease characterized by inflammation, including Ulcerative Colitis (UC), Crohn’s Disease (CD), and IBD-Unspecified (IBD-U). During the early 21st century, the incidence of pediatric IBD (pIBD) has demonstrated a significant upward trend. The reported incidence of pIBD and very early-onset IBD (VEOIBD) in Asia ranges from 0.5–21.6 to 0.2–1.4 per 100,000 person-years, respectively.^1^ Anti-TNFα biologics constitute the cornerstone first-line biological therapy for IBD patients.^2^, ^3^, ^4^ Nevertheless, VEOIBD exhibits greater treatment refractoriness, with 15% of patients demonstrating steroid non-response and 30% becoming steroid-dependent. Within two years of diagnosis, up to 50% of pIBD patients require escalation to biologic therapy.^5^, ^6^, ^7^ Real-world evidence demonstrates significantly reduced remission rates and elevated infliximab discontinuation in VEOIBD compared to older pediatric cohorts,^6^^,^^8^ infliximab induction frequently fails to achieve target therapeutic levels in pediatric CD, and primary non-response to adalimumab approaches 33% in VEOIBD, with nearly half of patients discontinuing therapy due to secondary loss of response within two years.^6^^,^^9^ This evidence collectively highlights a compelling therapeutic gap in this vulnerable population.

Thalidomide, initially marketed to treat morning sickness in pregnancy but withdrawn worldwide in the 1960s due to teratogenicity, has undergone clinical reevaluation. Its resurgence is attributed to demonstrated therapeutic efficacy in refractory diseases such as systemic lupus erythematosus and multiple myeloma, mediated through robust immunomodulatory mechanisms including TNF-α suppression and anti-angiogenic effects.^10^^,^^11^ These properties now support its application in adult and pIBD management. The limitations of biologic therapies in VEOIBD, particularly for biologic-refractory cases, make thalidomide a crucial rescue therapeutic option.^12^

However, Thalidomide-induced Peripheral Neuropathy (TiPN), which incidences of 27%–45% in pIBD, remains a major dose-limiting toxicity. It affects sensory, motor, and autonomic functions, exerting long-term detrimental effects on functional status, academic performance, and quality of life in children, contributing significantly to treatment discontinuation and compromised adherence.^13^, ^14^, ^15^ Current knowledge of TiPN primarily derives from studies in adults with multiple myeloma, its risk factors and clinical course remain poorly understood in pIBD patients.^16^ Moreover, pediatric studies report conflicting results on the cumulative dose-neurotoxicity relationship: some found no significant association, whereas long-term follow-up studies identified a correlation between high cumulative doses and overall adverse events.^13^^,^^17^ Given the critical therapeutic value of thalidomide in pIBD, especially for refractory CD and monogenic forms, the dose-toxicity relationship is clinically essential to guide optimal dosing regimens.

The primary aim of this study was to investigate the correlation between cumulative thalidomide dose and the incidence of TiPN in pIBD patients, with the goal of establishing a safe dosage threshold. Secondary aims included the assessment of clinical remission and adverse event rates.

## Materials and methods

### Study population

We conducted a retrospective cohort study at a single center belonging to Shenzhen Children's Hospital, covering the period from May 1, 2018, to April 30, 2025. Patients aged 0‒17 years with a diagnosis of IBD were identified using the Porto criteria.^18^

Thalidomide was administered at 1.0‒1.5 mg/kg/day. Patients could continue taking other medicines while on thalidomide. Dose adjustment or discontinuation due to peripheral neuropathic symptoms was decided by the pediatric gastroenterologist in consultation with a neurologist, based on neurological impairment severity and IBD activity. Follow-up duration was measured from the start of thalidomide until the last hospital visit. Those with insufficient treatment time were labeled as censored and not counted as responders when calculating response rates at specific times. Cumulative dose accounted for different courses of thalidomide without the intervals.

### Data collection and variables

Demographic and clinical variables included age, sex, IBD subtype (CD, UC, and IBD-U), disease distribution per Paris classification, previous and concurrent medications, treatment response, and electrophysiological findings. Laboratory parameters comprised Erythrocyte Sedimentation Rate (ESR), C-Reactive protein, White Blood Cell (WBC), hemoglobin and albumin.

### Assessment of efficacy and adverse events

For clinical evaluation of TiPN, we applied the National Cancer Institute Common Terminology Criteria for Adverse Events Sensory Scale (version 5.0, 2017). Thalidomide dosing data were collected from patient medical records, and Electromyography (EMG) was performed at baseline and every 6-months during follow-up, with additional assessments when patients developed symptoms or signs suggestive of peripheral neuropathy, enabling a focused analysis of how cumulative exposure correlates with peripheral neurotoxicity.

Treatment efficacy was assessed after one year of thalidomide therapy. Clinical remission was defined as a Pediatric Crohn’s Disease Activity Index (PCDAI) or Pediatric Ulcerative Colitis Activity Index (PUCAI) score below 10 after 12 months of thalidomide treatment. Mucosal healing was characterized by a score below 2 on the Simple Endoscopic Score for CD patients, or a zero on the Mayo endoscopic score. ESR of < 20 mm/h, and a CRP level of < 5 mg/L was defined as normal values.

### Statistical analysis

Categorical variables are presented as frequencies and percentages. Continuous variables are summarized as either median with Interquartile Range (IQR) or mean with Standard Deviation (±SD), based on their distribution. To evaluate potential confounding between the VEOIBD and pIBD groups, 1:1 propensity score matching based on age, sex, and IBD type was performed only to examine baseline covariate balance, rather than to generate a matched cohort for the final analysis. As all standardized mean differences were below 0.1, suggesting adequate balance between groups, all subsequent comparative analyses were performed in the full unmatched cohort (pIBD, n = 35; VEOIBD, n = 14) to maximize statistical power (Fig. S1).

Group comparisons were conducted using the Mann-Whitney *U test*, independent *t*-test, or Chi-Square test. Factors associated with TiPN were identified using logistic regression.

Receiver Operating Characteristic (ROC) curve analysis was employed to evaluate the predictive performance of cumulative thalidomide dose for TiPN; an Area Under the Curve (AUC) greater than 0.60 was considered clinically meaningful. Sensitivity and specificity were calculated at the optimal cutoff value. The difference in the area under the ROC curve between the univariate (dose-only) model and the multivariate (dose, gender, and age) model was compared using DeLong's test, with a significance level set at α = 0.05.

All statistical analyses and graphical work were performed with SPSS (version 26.0), R (version 4.3.0), and MATLAB. A two-tailed p-value < 0.05 was considered statistically significant.

## Results

### Population characteristics

A total of 49 patients met the inclusion criteria and were included in the final analysis. Four additional patients were excluded: one due to non-adherence and three due to insufficient follow-up data. All patients had initiated thalidomide therapy between May 1, 2018, and April 30, 2025. Baseline demographic and clinical characteristics are summarized in Table 1, and no concomitant neurotoxic medications were identified in either group.

**Table 1:** Patient characteristics.

	TiPN (n = 13)	No-TiPN (n = 36)	p-value
**Sex**			0.09
Male	10 (76.9)	18 (50%)	
Female	3 (23.1)	18 (50%)	
**Age**	14 (11, 16)	12 (6.75, 15)	0.18
Weight z score	0.36 (−1.71, 1.09)	0.42 (−2.55, 1.03)	0.95
Height z score	0.57 (−1.34, 2.27)	0.05 (−1.83, 1.55)	0.93
**IBD type**			0.82
Crohn’s disease	11 (84.6)	29 (80.6)	
Ulcerative colitis	0	1 (2.8)	
IBD-U	2 (15.4)	6 (16.7)	
**Crohn’s disease**^a^			0.85
L1	2 (15.4)	5 (13.9)	
L2	3 (23.1)	6 (16.7)	
L3	5 (38.5)	19 (52.8)	
L4a	1 (7.7)	1 (2.8)	
L4	4 (30.8)	12 (33.3)	
**Ulcerative colitis**^b^**and IBD-U**			
E1	0	1 (2.8)	
E2	0	0	
E3	0	0	
E4	0	0	
**Previous treatment**			0.84
Steroids	3 (23.1)	10 (27.8)	
Exclusive enteral nutrition	4 (30.8)	14 (38.9)	
Aminosalicylates	9 (69.2)	22 (61.1)	
Azathioprine	0	1 (2.8)	
Methotrexate	0	2 (5.6)	
Infliximab	6 (46.2)	17 (47.2)	
Adalimumab	0	1 (2.8)	
Ustekinumab	0	1 (2.8)	
**Duration before thalidomide, mon**	14 (6, 25)	15 (5.5, 24.25)	0.87
**Follow-up duration, months, median (IQR; range)**	16 (13.5‒21.5; 3‒27)	10 (6‒18; 3‒49)	0.054
**Concomitant neurotoxic medications**	0 (0)	0 (0)	NA
**Active fistula/abscess**			0.43
1	2 (15.4)	12 (33.3)	
0	10 (76.9)	23 (63.9)	
**Previous surgeries**			0.43
1	2 (15.4)	12 (33.3)	
0	10 (76.9)	23 (63.9)	
**ESR**	22 (5, 32)	28 (13, 48)	0.29
**C-Reactive protein**	6.4 (0.53, 19.8)	7.76 (2.05, 20.7)	0.71
**Albumin**	38 (34, 43)	37.8 (34.83, 40.78)	0.57
**Hemoglobin**	116.08 ± 16.34	116.58 ± 14.37	0.92
**Platelets**	361.85 ± 146.19	377.87 ± 124.14	0.71
**White blood cell (10^9^/L)**	7.93 (7.04, 10.74)	8.23 (6.92, 10.71)	0.75
**Baseline PCDAI**^c^	22.0 (10.5, 25.8)	20.0 (12.0, 24.2)	0.88

Values are presented as n (%) or median.

IBD, Inflammatory Bowel Disease; IBD-U, Inflammatory Bowel Disease Unclassified; ESR, Erythrocyte Sedimentation Rate.

a L1: Distal 1/3 ileum ± limited cecal disease; L2: Colonic disease; L3: Ileocolonic disease; L4a: Upper disease proximal to ligament of Treitz; L4b: Upper disease distal to the ligament of Treitz and proximal to the distal 1/3 ileum; L4ab: Upper disease involving both L4a and L4b.

b E1: ulcerative proctitis; E2: left-sided UC (distal to splenic flexure); E3: extensive (distal to hepatic flexure); E4: pancolitis (proximal to hepatic flexure).

c Among patients with Crohn’s disease and available baseline PCDAI data only (TiPN: n = 9; No-TiPN: n = 21). PUCAI was not compared because only one patient had ulcerative colitis.

As shown in Table 1, TiPN occurred in 13 patients (26.5%). Among these, 6 (46.2%) exhibited both clinical symptoms/signs and electrophysiological abnormalities, 3 (23.1%) had subclinical neuropathy identified only through instrumental studies, and 4 (30.8%) reported neuropathic symptoms without corresponding electrophysiological changes. Of the patients with both clinical and electrophysiological TiPN, nerve conduction abnormalities preceded clinical symptoms in 4 (66.7%), occurred concurrently in 1 (16.7%), and appeared after symptom onset in 1 (16.7%).

Comparative analysis between patients with and without TiPN revealed no significant associations with age at diagnosis, sex, IBD type and location, surgical history, disease duration before thalidomide initiation, or previous treatments and their responses. Additionally, none of the baseline laboratory parameters ‒ including ESR, CRP, albumin, hemoglobin, platelets, white blood cell ‒ were significantly associated with the development of TiPN.

### Comparison of baseline characteristics between VEOIBD and pIBD

Propensity score matching improved the balance of the major covariates (Sex, Age, IBD type) between the groups. Following matching, the standardized mean differences for all variables fell below the 0.1 threshold (Fig. S1), indicating adequate balance for subsequent analysis.

Among the enrolled cohort, 14 patients were diagnosed with VEOIBD. Thalidomide therapy was initiated before the age of 6 in 11 VEOIBD patients and between ages 6 and 9 in 3 patients. Baseline clinical characteristics are presented in Table S1. One patient in the pIBD group had IBD-U.

Colonic disease location was predominant in VEOIBD patients with CD, whereas ileocolonic involvement was more frequently observed in the pIBD group. The majority of patients in both groups had treatment-refractory disease and a history of resistance to multiple therapies. Twelve VEOIBD patients (85.7%) and 8 pIBD patients (22.9%) were not exposed to biologics (Table S1).

Notable differences were observed in treatment history: biologic agents formed the cornerstone of previous treatment in pIBD patients, whereas corticosteroids were more commonly used in the VEOIBD group, indicating distinct therapeutic profiles between the two cohorts.

### The predictive value of cumulative dose for TiPN

**Fig. 1:**
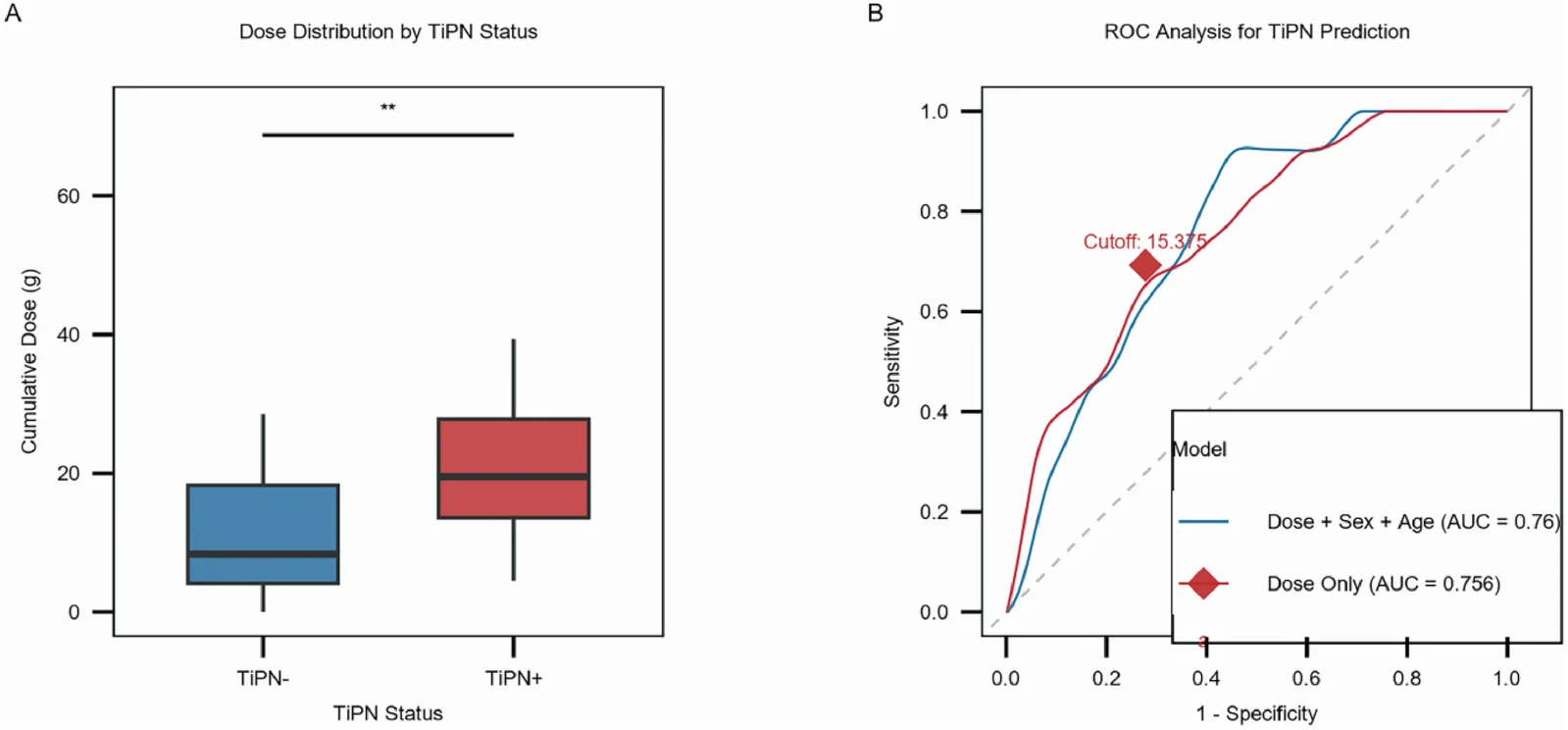
Cumulative dose dependence and predictive value of Thalidomide-induced Peripheral Neuropathy (TiPN). (A) TiPN associated with cumulative dose during the treatment with thalidomide (**p < 0.01). (B) Receiver operating characteristic curve analysis for TiPN Prediction.

TiPN was observed in 13 of 49 patients (26.5%). As shown in Fig. 1A, the median cumulative thalidomide dose was significantly higher in the TiPN group than in the non-TiPN group (19.5 *g* vs. 8.35 g; p = 0.008). Fig. 1B shows the standard (cumulative dose) vs. covariate-adjusted ROC curves. In the standard ROC curve constructed using cumulative dose, we established the optimal threshold for the cumulative thalidomide dose in predicting TiPN. The AUC was 0.756 (95% Confidence Interval [95% CI] 0.608‒0.905). We demonstrated that a cumulative thalidomide dose level > 15.38 g was the optimal cutoff for detecting TiPN, with a sensitivity of 0.722, and a specificity of 0.692. Besides, we mitigated the influence of inter-patient differences by adjusting for sex and age as covariates, enhancing the robustness of our conclusions. The ROC curves of the standard group and the covariate-adjusted group show similar AUC values (0.756 [0.608, 0.905] vs. 0.76[0.62, 0.899]). Consequently, the standard AUC proves to be a reasonable metric for assessing performance in this context.

### Efficacy

**Fig. 2:**
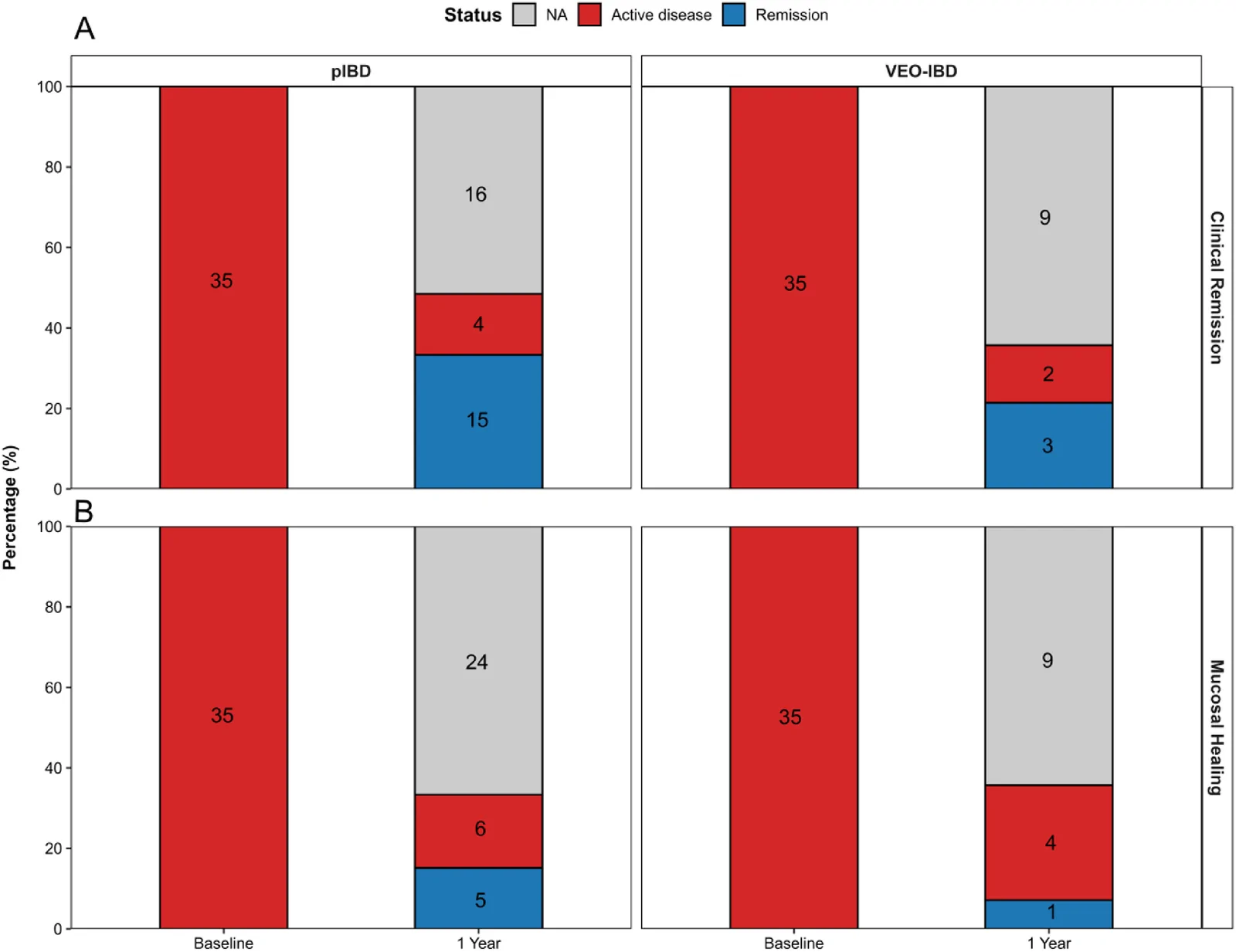
Rates of clinical remission and mucosal healing in Very Early-Onset (VEOIBD) and pediatric-onset (pIBD) inflammatory bowel disease. (A) Clinical remission; (B) Mucosal healing. Not Available (NA) refers to patients lacking assessment due to an unevaluated time point or missed endoscopy.

During the one-year follow-up, 18 (75%) of the 24 IBD patients maintained clinical remission (Fig. 2A). The remission rate was 78.9% (15/19) in the pIBD group and 60% (3/5) in the VEOIBD group. Among those not in remission at one year, all had only mild disease activity. Mucosal healing was achieved in 6 of 16 (37.5%) patients overall, including 1 of 5 (20%) in the VEOIBD subgroup and 5 of 11 (45.5%) in the pIBD subgroup (Fig. 2B). Compared with the VEOIBD group, patients in the pIBD group showed numerically higher rates of clinical remission and mucosal healing at one year; however, these differences did not reach statistical significance.

**Fig. 3:**
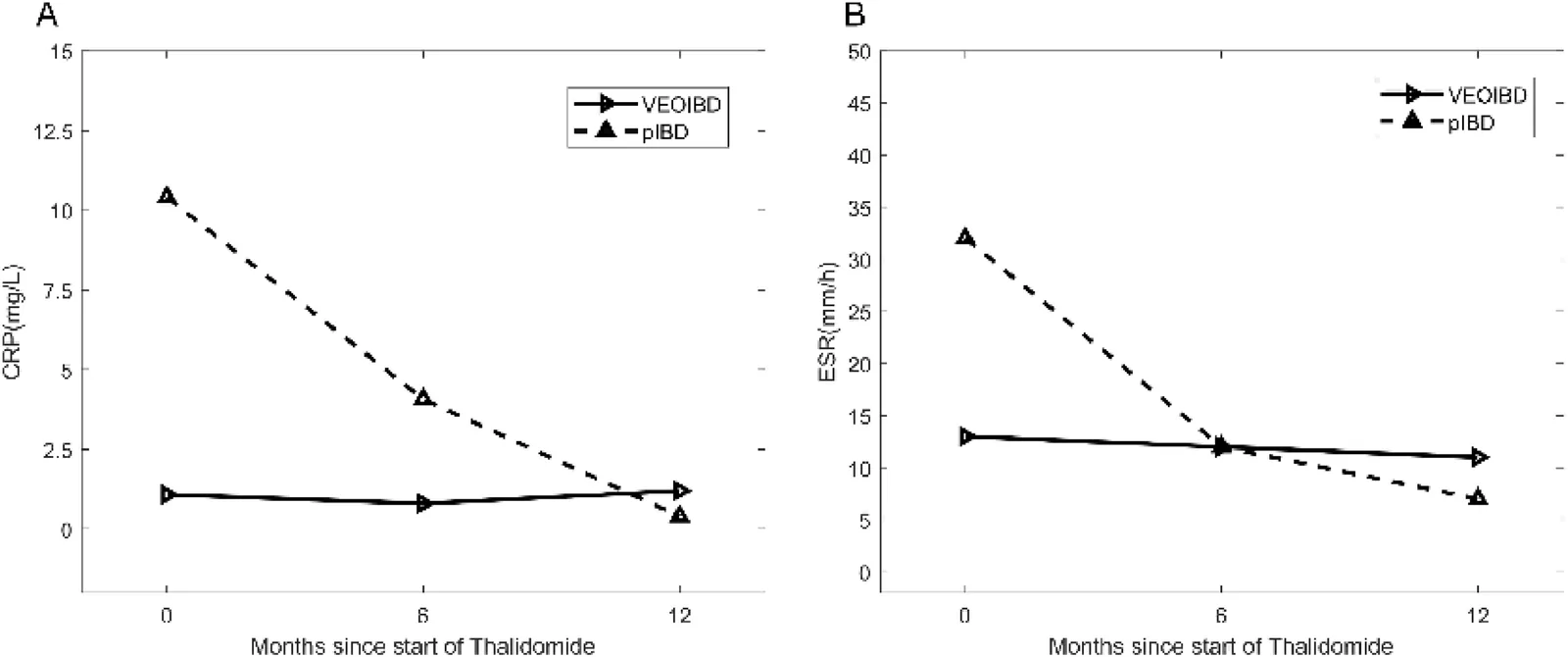
Trends of CRP and ESR levels over time in the 2 groups of patients. (A) C-Reactive Protein (CRP); (B) Erythrocyte Sedimentation Rate (ESR).

In the first year of treatment, the medians of the CRP and ESR levels decreased with a similar trend in the two groups (Fig. 3A‒B). At baseline, the pIBD group exhibited a significantly higher median CRP level (p = 0.005) compared to the VEOIBD group, whereas the median ESR level was higher but did not reach statistical significance (p = 0.09) (Table S1).

### Tolerance

Compared to patients with pIBD, a significantly lower number of patients with VEOIBD experienced Adverse Events (AEs) (3/14 vs. 19/35, p = 0.04). The types of AEs are summarized in Table 2. All cases of rash, drowsiness or fatigue resolved completely. Peripheral neuropathy resolved in 4 patients, while outcomes remained unknown for 3 others at follow-up.

**Table 2:** Comparison of adverse drug reaction profiles.

Characteristics	pIBD (n = 35)	VEOIBD (n = 14)	p-value
Rash	5 (14.3%)	0 (0%)	0.31
Drowsiness	3 (8.6%)	0 (0%)	0.55
Fatigue	3 (8.6%)	0 (0%)	0.55
Peripheral neuropathy	10 (28.6%)	3 (21.4%)	0.73
Total adverse events	19 (54.3%)	3 (21.4%)	0.04

pIBD, Pediatric-onset Inflammatory Bowel Disease; VEOIBD, Very Early-Onset Inflammatory Bowel Disease.

As shown in Table 3, treatment discontinuation due to AEs occurred in 1 and 7 patients in the VEOIBD and pIBD groups, respectively (p = 0.18).

**Table 3:** Reasons for treatment discontinuation.

Reason	VEOIBD	pIBD	p-value
Adverse events	1	7	0.18
Poor compliance	0	6	NA
Relapse	1	2	NA
Prolonged remission	0	0	NA
Other reasons	0	1	NA

pIBD, Pediatric-onset Inflammatory Bowel Disease; VEOIBD, Very Early-Onset Inflammatory Bowel Disease; NA, Not Applicable. The small sample size in the VEOIBD group likely resulted in insufficient statistical power.

## Discussion

In this retrospective cohort study, we demonstrate that cumulative thalidomide dose strongly predicts the development of TiPN in children, with a cutoff value of > 15.38 g offering clinically meaningful predictive utility. More notably, we observed that patients with VEOIBD ‒ despite exhibiting more severe and treatment-refractory disease ‒ experienced significantly fewer overall adverse events than those with later-onset IBD, suggesting a distinct tolerability profile in this clinically challenging subgroup. Our study confirms that thalidomide demonstrates significant efficacy and a favorable tolerability profile in pediatric patients with pIBD and VEOIBD who are refractory to standard therapies. Approximately half of the patients achieved clinical remission within one year, a finding consistent with the results reported by Bramuzzo et al.^12^

The AUC was 0.756 for the dose-only model and 0.76 for the covariate-adjusted model, indicating comparable discriminative ability between the two approaches. This minimal improvement in AUC (0.004) with the addition of sex and age variables suggests that cumulative dose remains the predominant predictor of TiPN risk, while demographic factors contribute only marginally to risk stratification.

Although cumulative dose has been proposed as a potential risk factor for thalidomide adverse events, this association lacks consistent support.^19^^,^^20^ One study in children and adolescents observed no cases of peripheral neurotoxicity at cumulative doses below 28 g^21^; another proposed a risk threshold of 20 g^22^; and research from a U.S. group reported that all patients receiving cumulative doses exceeding 60 g developed neuropathy.^19^ Other studies suggested that the risk of TiPN is associated with mean daily dose and influenced by single nucleotide polymorphisms in the ICAM1, SERPINB2, and BDNF genes.^23^^,^^24^ Against this backdrop of inconsistent findings, our study strengthens the evidence by clearly establishing cumulative thalidomide exposure as a critical determinant of TiPN. Moreover, it is the first to identify a well-defined and clinically applicable dose threshold (> 15.38 g) within a pediatric IBD cohort. The longer follow-up duration observed in the TiPN group suggests that treatment exposure time may have contributed, at least in part, to the higher cumulative dose in these patients. Therefore, the association between cumulative dose and TiPN should be interpreted in the context of exposure duration.

The cumulative risk dose identified in our analysis is lower than those previously reported, which may be explained by several factors. First, as our study focused exclusively on Chinese participants under the age of 18, this discrepancy may be attributed to ethnicity- and age-related variations in drug metabolism. Second, given the off-label use of thalidomide, our center adhered to more conservative dosing guidelines, resulting in lower median doses compared to those typically administered to adults. These findings underscore the necessity of establishing age- and ethnicity-specific dosing thresholds for predicting toxicity in pediatric populations receiving thalidomide.

In this study, neurophysiological assessment was employed to detect early and even subclinical neuropathic changes. Nearly half of the patients exhibited both clinical symptoms and electrophysiological abnormalities. Notably, in the majority of cases (66.7%), electrophysiological alterations preceded the onset of clinical symptoms, suggesting that electrophysiological changes may serve as a more sensitive indicator of TiPN than subjective reports ‒ a finding consistent with previous studies.^25^ On the other hand, 30.8% of patients presented with neuropathic symptoms in the absence of electrophysiological abnormalities. This discrepancy may suggest the presence of small fiber neuropathy, undetectable by conventional electromyography, or alternatively reflect the non-specific nature of certain neuropathic symptoms, consistent with the predominantly sensory involvement characteristic of TiPN.^26^^,^^27^

The clinical implications of our findings are substantial. Earlier reports indicated that only 19%–36% of VEOIBD patients on infliximab continued treatment beyond 12-months. Remission rates at one year were also low: 10%–11% for CD, 7%–25% for UC, and nearly absent in IBD-unclassified.^28^^,^^29^ By comparison, all children in our cohort began thalidomide during active disease. After 12-months, over half of the patients had achieved clinical remission, and nearly 50% remained on the drug. Importantly, only one VEOIBD patient discontinued due to TiPN, highlighting the promising efficacy and acceptable tolerability of thalidomide in pIBD.

This study has several limitations. Its retrospective nature and small sample size limited full assessment of dosing strategies and reduced statistical power. The small sample size may affect the generalizability and stability of the model estimates. Moreover, the relatively wide confidence intervals for the AUCs (0.608–0.905 for the dose-only model; 0.620–0.899 for the combined model) reflect uncertainty in the point estimates. External validation using larger, multi-center cohorts is needed to confirm these findings and refine cutoff values across diverse populations. Furthermore, because of the retrospective nature of the study, standardized disease activity scores were not uniformly documented for all patients.

Since some patients continued thalidomide despite electrophysiological abnormalities, the possibility that maintaining the dosage could worsen TiPN cannot be ruled out. Comparisons between VEOIBD and older groups were complicated by inherent disease differences. We used statistical matching to address this, but residual confounding may remain due to limited samples. The duration of instrumental follow-up after thalidomide discontinuation was limited. Although most symptoms were transient, longer neurological assessment is needed to evaluate long-term TiPN outcomes and adult neuropathy risk. So far, no new neurological symptoms have been observed.

Despite these limitations, our findings carry immediate clinical relevance. Vigilant monitoring of cumulative thalidomide exposure is imperative, and electrophysiological assessment should be considered once cumulative dose exceeds 15 g ‒ particularly in children requiring extended therapy. The favorable overall tolerability in VEOIBD supports the ongoing use of thalidomide in this difficult-to-treat population, though careful attention to neurological sequelae remains essential.

In conclusion, we identify cumulative thalidomide dose as a key predictor of TiPN in children with IBD and propose a clinically actionable cutoff for risk stratification. The reduced non-neurological toxicity observed in VEOIBD patients may broaden the therapeutic window for thalidomide in this severe disease subset. Future studies integrating pharmacological monitoring and genetic biomarkers are needed to develop personalized treatment strategies that maximize efficacy while minimizing toxicity in vulnerable pediatric populations.

## Conclusions

Our findings establish that cumulative drug dose is a key determinant of TiPN in pIBD. This dose-dependent neurotoxicity underscores that vigilant monitoring of the cumulative dose is a critical strategy for optimizing the therapeutic index of thalidomide. Intriguingly, patients with VEOIBD, a typically more treatment-refractory cohort, demonstrated a paradoxically favorable tolerability, suggesting a unique risk-benefit consideration within this specific subgroup.

## Ethical considerations

This retrospective cohort study was conducted in accordance with the ethical principles of the Declaration of Helsinki, approved by the Ethics Committee of The Shenzhen Children’s Hospital (KY202500102), and reported following the STROBE (Strengthening the Reporting of Observational Studies in Epidemiology) guidelines.

## Consent for publication

Not applicable.

## Data availability

The datasets generated and/or analyzed during the current study are available from the corresponding author upon reasonable request.

## Declaration of generative AI and AI-assisted technologies

AI-assisted tools were used solely for English language editing during manuscript preparation. All content was reviewed and revised by the authors, who bear full responsibility for the accuracy and integrity of the final manuscript.

## Authors’ contributions

All authors participated in the design, interpretation of the studies, data analysis, and manuscript review. JM drafted the work; PL and ZQ C were responsible for data acquisition; SL was responsible for data analysis; LY and ZB C proofread patient information; ZX W oversaw the study design. All authors read and approved the final manuscript. ZY L assisted in reviewing and verifying the use of combination medications within the study cohort.

## Declaration of competing interest

The authors declare no conflicts of interest.

## Appendix Supplementary materials

Image, application 1
