# Supplementary material for: Cumulative thalidomide exposure, peripheral neuropathy, and clinical outcomes in pediatric and very early-onset inflammatory bowel disease: a retrospective cohort study
Source: Clinics (Sao Paulo). 2026 Sep 8;81:101093. doi: 10.1016/j.clinsp.2026.101093 (PMC13578953; doi:10.1016/j.clinsp.2026.101093)
Supplement: Supplementary file 1 [file mmc1.docx]

CLINICS-D-25-02090_Supplementary Material

**Table S1** Patient characteristics

|  | **pIBD (n = 35)** | **VEOIBD (n = 14)** | **p-value** |
| --- | --- | --- | --- |
| **Age, median (IQR)** | 15 (12, 16) | 3.5 (2, 5.75) | <0.001 |
| **IBD type** |  |  | <0.0001 |
| Crohn’s disease | 32 (90) | 6 (40) |  |
| Ulcerative colitis | 0 | 1 (7) |  |
| IBD-U | 1 (3) | 7 (50) |  |
| **Duration before thalidomide (month)** | 16 (6, 26) | 12 (4.5, 15.75) | 0.25 |
| **Localization** |  |  |  |
| Crohn’s diseasea^a^ |  |  | 0.008 |
| L1 | 4 (11.4) | 1(7.1) |  |
| L2 | 3 (8.6) | 4 (28.6) |  |
| L3 | 22 (62.9) | 1 (7.1) |  |
| L4a | 2 (5.7) | 0 |  |
| L4 | 15 (42.9) | 0 |  |
| Ulcerative colitis^b^ and IBD-U |  |  |  |
| E1 | 0 | 1 (7.1) |  |
| E2 | 0 | 0 |  |
| E3 | 0 | 0 |  |
| E4 | 0 | 0 |  |
| **Previous treatment** |  |  | 0.05 |
| Steroids | 5 (14.3) | 8 (57.1) |  |
| Exclusive enteral nutrition | 16 (45.7) | 4 (28.6) |  |
| Aminosalicylates | 19 (54.3) | 7 (50) |  |
| Azathioprine | 1 (2.9) | 0 |  |
| Methotrexate | 0 | 2 (14.3) |  |
| Infliximab | 21 (6) | 6 (42.9) |  |
| Adalimumab | 1 (2.9) | 0 |  |
| Ustekinumab | 1 (2.9) | 0 |  |
| **Active fistula/abscess, n (%)** |  |  | 0.01 |
| Yes | 14 (40) | 0 |  |
| No | 19 (54.3) | 14 (100) |  |
| **Concomitant treatment** |  |  | 0.42 |
| Steroids | 3 (8.6) | 2 (14.3) |  |
| Partial enteral nutrition | 2 (5.7) | 0 |  |
| Aminosalicylates | 19 (54.3) | 4 (28.6) |  |
| Azathioprine | 2 (5.7) | 0 |  |
| Infliximab | 23 (65.7) | 5 (35.7) |  |
| Adalimumab | 2 (5.7) | 0 |  |
| **Drug Withdrawal, n (%)** |  |  | 0.04 |
| Yes | 16 (45.7) | 2 (14.3) |  |
| No | 19 (54.3) | 11 (78.6) |  |
| **ESR** | 32 (91.4) | 13 (92.9) | 0.09 |
| **C-Reactive protein** | 10.44 (5.65, 23.6) | 1.09 (0.43, 7.9) | 0.005 |
| **Albumin** | 37.15 (33.7, 39.6) | 40.8 (38, 42.1) | 0.07 |
| **Hemoglobin** | 115.5 (110, 123.75) | 110 (106.25, 119.25) | 0.17 |
| **Platelets** | 364.86 ± 135.67 | 390.86 ± 117.97 | 0.54 |
| **White blood cell (10^9^/L)** | 7.93 (6.59, 11.26) | 8.36 (7.71, 10.67) | 0.61 |
| **Baseline PCDAI^c^** | 20.0 (12.0, 24.5) | 24.2 (18.1, 26.2) | 0.41 |

Values are presented as n (%) or median (interquartile range).

IBD, Inflammatory Bowel Disease; IBD-U, Inflammatory Bowel disease Unclassified; ESR, Erythrocyte Sedimentation Rate.

^a^ L1: Distal 1/3 ileum ± limited cecal disease; L2: Colonic disease; L3: Ileocolonic disease; L4a: Upper disease proximal to ligament of Treitz; L4b: Upper disease distal to the ligament of Treitz and proximal to the distal 1/3 ileum; L4ab: Upper disease involving both L4a and L4b.

^b^ E1: Ulcerative proctitis; E2: Left-sided UC (distal to splenic flexure); E3: Extensive (distal to hepatic flexure); E4: Pancolitis (proximal to hepatic flexure).

^c^ Among patients with available baseline PCDAI data only (pIBD: n = 25; VEOIBD: n = 5). p-value was calculated using the Mann-Whitney *U*-test.

**Figure S1** Love Plot illustrating the balance of covariates before and after propensity score matching (PSM). Covariates include Sex, IBD_type, and Age.
